# Stereo Vision-Based Human–Robot Interaction for Weld Seam Detection in Robotic Welding

**DOI:** 10.3390/s26154841

**Published:** 2026-07-31

**Authors:** Pushkar Kadam, Gu Fang, Farshid Amirabdollahian, Ju Jia Zou, Patrick Holthaus

**Affiliations:** 1 Centre for Advanced Manufacturing Technology, School of Engineering, Western Sydney University, Locked Bag 1797, Penrith, NSW 2751, Australia; j.zou@westernsydney.edu.au; 2 Robotics Research Group, University of Hertfordshire, Hatfield AL10 9AB, UK; f.amirabdollahian2@herts.ac.uk (F.A.); p.holthaus@herts.ac.uk (P.H.)

**Keywords:** human–robot interaction, computer vision, robotic welding, stereo vision, deep learning, hand detection

## Abstract

Robotic welding has been widely used to improve manufacturing efficiency. However, for small batches or one-off jobs, the time and effort required for robotic welding may be economically prohibitive. An intuitive ‘mimicking’-like robotic welding approach can help non-robotics experts instruct the robot to perform the welding operations by letting them demonstrate the welding path via hand gestures without requiring any programming. In this paper, we present a human–robot interaction (HRI)-based weld seam detection for such a robotic welding application. Our approach consists of a vision-based hand detection and tracking method for the user to demonstrate the welding path to the robot’s vision system. We then isolated the welding seam lines and developed a search algorithm to identify the welding paths for the robot. The continuous weld seam path is observed within the bounds of the seam edge in the image and is projected to the robot coordinate space. The seam detection is thoroughly tested with a real-world application on the UR10e robot. The evaluation has revealed an accuracy of 1 pixel in the image plane, equivalent to 1 mm in physical space in our setup.

## 1. Introduction

The shift in manufacturing technologies toward Industry 5.0 [1] has led to the development of processes that enable humans to collaborate with machines. For autonomous collaborative robots (*Cobots*), it is important that individuals without a robotics background can operate them without programming experience [2]. These individuals, known as non-robotics experts [3], are experts in their respective fields who use robots as tools to automate their tasks. These tasks range from robotic surgery to various manufacturing-related operations [1]. Given the wide range of robotics applications by non-robotics experts, it is important to develop novel methods that enable such users to perform tasks without having to program the robot. This type of automation enables non-robotics experts to increase their productivity and can be implemented in manufacturing processes such as robotic welding.

Demonstration methods have been widely adopted in robotics, enabling users to instruct robots to perform specific tasks without programming [4]. These methods are similar to instructing another human to perform a task, and they also enable non-robotics experts to instruct robots using computer vision [5], teleoperation [6], and large language models [1]. Computer vision methods observe the user while demonstrating an action and then imitate it on the robot platform [3]. These demonstrations often take the form of gestures, such as sign language [5], object tracking [7], or hand-tracking movements [8]. One advantage of using computer vision is that it does not require other types of robot interface devices.

Robotic welding is an important operation in manufacturing. For large-scale, repetitive tasks in controlled environments, robots have been successfully used to perform welding tasks [9,10]. Additional sensors, such as active light [11,12,13,14] or passive computer vision [15,16,17,18] can be used to guide the robot to the correct path. Furthermore, for one-off or small batch-sized weldments, constantly programming the robot becomes uneconomical for robotic welding. Therefore, it is important to automate seam detection in robotic welding to improve the productivity of the welding operation.

### 1.1. Robot Vision-Based Systems

Computer vision has become prominent in robotics applications as it helps robots to perceive their surroundings. The ability to perceive their environment with different types of sensors enables autonomous robots to plan their operations. Developing computer vision-based methods is helpful for human–robot collaboration because humans and robots can share the same concept of their work environment [19]. Different types of autonomous robotic welding applications incorporate computer vision to detect the weld seam line [15,16,17,18]. Computer vision can be used to detect weld seams as perceived by humans, which is beneficial for non-robotics experts [3]. Therefore, in human–robot interaction (HRI), computer vision systems have become important for creating a common ground for humans and robots regarding their environment.

The type of computer vision used with autonomous robots relies on the application. The camera selection process is an important first step in the robot method development, and the cameras are usually classified as monocular, stereo, and RGB-D (Red, Green, Blue-Depth) cameras. Monocular cameras are widely used in robotics applications, but they lack depth estimation capabilities [7]. A stereo camera combines two monocular cameras and often replicates human binocular vision for depth estimation [16,20]. RGB-D cameras use infrared light to provide the colour channels (RGB) along with an additional depth channel (D) [21,22]. Each camera type requires calibration to convert image points to camera coordinates [23,24]. Therefore, it is important to select the appropriate camera and calibration process for robotic applications.

In robotic applications using computer vision, it is important to determine the positions of objects in the robot’s coordinate space. Robot hand–eye calibration allows the robot to transform the object from the camera’s image plane to the robot’s coordinate system. Hand–eye calibration methods are divided into two categories: hand-to-eye (camera mounted away from the robot) [24,25] and hand-in-eye (camera mounted on the robot) [26,27]. This calibration process is the preliminary step before using computer vision in robotics and must be performed if there is any change in the robot and camera setup.

### 1.2. Vision-Based Seam Detection in Robotic Welding

Weld seam detection using computer vision for robotic welding involves first capturing an image of the weldment and processing it to detect the weld seam. While the robot path-following method will depend on the type of vision sensor used, the image processing steps are likely to be similar for an RGB camera. In addition to active and passive vision methods, deep learning methods have also been developed for weld seam detection [28,29]. Once the seam lines are detected, the robot path for the welding operation is planned. Effectiveness is measured either by visual evaluation of the welding path [15,17] or by measuring the pixel range [30] in the image plane. Furthermore, after detecting the path in the image plane, the seam path image coordinates must be converted to robot coordinates for the robot implementation. Therefore, whichever approach is used, the result is always a sequential, continuous weld seam path for the robot to follow.

Active vision for seam detection involves using an auxiliary light source, such as a laser. These laser pointers are mobile and scan over the surface of the weld piece to detect the seam. Wang et al. [11] developed a method that uses laser light on weld seams and employs Probabilistic Neural Networks (PNNs) [31] to classify weld joint types. Structured light is also used to achieve multi-pass welding [12]. Active vision can also be used in the presence of heavy noise by using a laser light scanner and curve-fitting algorithms on the detected seam [13,14]. The advantage of the active vision approach is that the image processing techniques need only filter out bright laser reflections to identify the seam. Active vision is also useful for longer weld seam lines that would otherwise fall outside the camera’s field of view. The disadvantage of using active vision is that the user must scan the weld piece before the detection process. Furthermore, the robotic welding setup is stationary, where the robot workspace, camera position and laser scanner are pre-calibrated. Therefore, an active vision approach for seam detection is suitable for a stationary robotic welding setup in a controlled factory environment for longer seam lines.

In passive vision for seam detection, emphasis is placed on image processing techniques applied to captured images. The images are captured from a calibrated camera, and then different morphological operations are performed on the images until the weld seam pixel coordinates are obtained. Shi et al. [15] developed a passive vision-based approach for butt joint welding, which used different image morphological operations such as Canny edge detection [32] and Harris corner detector [33]. Dinham and Fang [16] implemented a stereo vision system for weld seam detection using Sobel edge detection [34] followed by A* search algorithm [35]. Wang et al. [18] improved upon Binary Robust Independent Elementary Features (BRIEF), called multi-BRIEF, in fillet weld detection that takes advantage of features such as scratches or dents on the weldment. Shah et al. [30] performed noise reduction followed by using Sobel edge detection [34] for seam detection. Most of these methods follow a similar approach: reducing noise by operating on binary images and developing a search algorithm to detect the continuous weld path for robot welding. The advantage of passive vision is that it has no moving parts, unlike laser scanners. However, the welding path is limited to the camera’s field of view. Therefore, the welding path can be determined by integrating image processing techniques and continuous weld seam path search algorithms.

Deep learning-based methods are also developed for robotic welding. Deep learning methods are usually based on seam bead recognition [20] and require an annotated image dataset for training. The weld seam is treated as an object detection problem, and deep learning methods such as YOLO (You Only Look Once) [36] have been used [20]. Xiao et al. [37] combined active light and a deep learning approach for seam line detection using R-CNN (Regional-Convolutional Neural Network) [38]. Jin et al. [39] used Mask R-CNN [40] by annotating the seam line with a segmentation mask on CAD (Computer Aided Design) data. Liu et al. [29] developed a method to recognise multiple weld paths that addresses the complexity of steel components and the welding environment by using a CNN together with feature detectors such as SIFT [41]. The deep learning approach requires training data, which is often not annotated along the weld seam. The models are often trained using transfer learning with data from other contexts [29] or a synthetic dataset [39], which may lack real-world complexity. Furthermore, the welding process may be relevant to the type of weld seams in the dataset, and the model may not generalise to different weld seam lines. Therefore, deep learning has applications in robotic welding for the known seam path types.

### 1.3. Human–Robot Interaction for Welding

A review of the literature shows that passive vision is more beneficial than active vision as there are no moving parts [16]. While deep learning approaches are investigated [20,37,39], they often rely on an annotated dataset. However, relying entirely on computer vision has problems, such as the weldment being located in a limited space [15], a designated workspace [16], and prior knowledge of the weldment type to implement the search [30]. Table 1 highlights some of the important papers relevant to our work. Therefore, relying on computer vision alone brings forth certain limitations that can be overcome with Human–Robot Interaction (HRI) [2].

Considering the limitations of the current computer vision-dependent process, as highlighted in Table 1, human intuition can assist the robot in carrying out repetitive tasks. The current limitations of a pre-defined search area, or prior knowledge of the weld piece to select the appropriate methods, can be overcome with the human–robot interaction approach. The human demonstration of the path can be specific to weld seam types, and the methods can be generalised to all weld types. Furthermore, human demonstrations will be beneficial for identifying composite weld seams and ensuring that false positives in weld seam detection on multiple weldment edges can be avoided.

Kinesthetic teaching in HRI involves human operators moving the passive joints of the robot with the desired motions [3]. While the latest *Cobots* used in welding are equipped with kinaesthetic teaching functionality, there is still a high reliance on teleoperation as per a recent review [42]. Gesture-based demonstrations using computer vision will help non-robotics experts demonstrate the welding path as required by the user. Therefore, HRI for robot welding applications with computer vision is an important process to ensure that the robot vision system identifies the correct weld seams.

### 1.4. Contributions

In this paper, we developed a human–robot interaction-based approach to identify the weld seam path for robotic welding using a stereo camera to observe human demonstration with finger pointing. We used the hand-pose detection methods developed in previous work [8] and developed a tracking-by-detection approach to track fingertips in real time with live rendering of hand pose to assist the user during demonstrations. Using the demonstrations, we developed methods to identify the weld seam by isolating the seam region and developed a weld search path algorithm. We also compared our developed search algorithm with A* search [35] to test robustness on the weld path detection. Using the hand-to-eye calibration process from our previous work [24], we were able to project the weld path from the image plane to the robot coordinate frame to simulate a welding path on the UR10e robot arm. The main contributions of this paper are:

1. Hand tracking-by-detection method for seam area localisation.
2. A weld seam detection method using image processing techniques.
3. An alternative seam search path algorithm to A* that identifies a continuous weld path for the robot welding that is also capable of backtracking to avoid getting stuck in spurs or edge offshoots.
4. Composite seam detection method that identifies multiple weld seams on a composition of weldments.

This paper is structured into five sections. Section 2 presents the different methods, including hand fingertip tracking, seam detection, weld path search, composite weld seam detection, and robot seam path implementation. Section 3 outlines the experimental setup and also presents the results for the seam path detection in both the image plane and the robot coordinate space. Section 4 presents a discussion of the method and results and elaborates on the strengths and limitations of the system. Section 5 concludes the paper and outlines future work.

## 2. Materials and Methods

**Figure 1:**
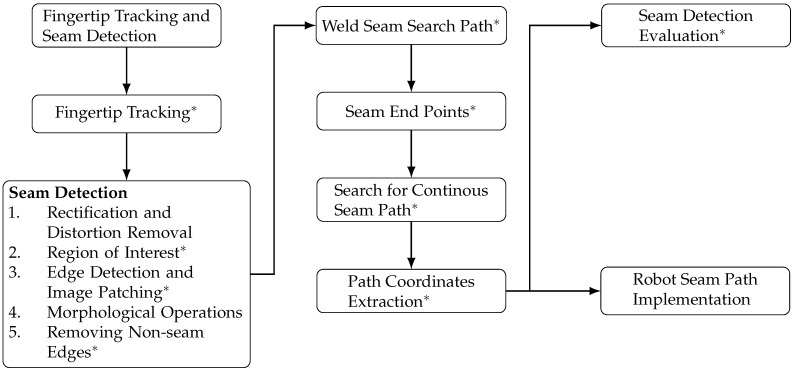
Flow chart of the weld seam detection process. (*) indicates novel methods developed in this paper. Section 2.4, which discusses ‘Composite Weld Seam Detection*’, is not included in the flowchart as it requires the steps from Section 2.1 and Section 2.2 for each seam in composite weld seam detection.

In this section, we present the methods developed for a human operator to indicate to the robot, using finger pointing, the weld seams to be welded. The flow chart of the approach is shown in Figure 1. Section 2.1 outlines the process of fingertip tracking to localise the weld seam, and different image processing techniques to detect the seam (Contributions 1 and 2). In Section 2.2, we present a search algorithm that computes a continuous weld seam path for robot implementation and backtracks to avoid getting stuck in local spurs or offshoots of the seam edge (Contribution 3). In Section 2.4, we illustrate the developed workflow that enables multiple seam line detections for composite welding (Contribution 4). In Section 2.5, we outline a detailed approach for weld path implementation on the robot while considering different constraints.

### 2.1. Fingertip Tracking and Seam Detection

In previous work [8], we developed an end-to-end deep learning method that can detect fingertips. In this paper, we first track the fingertip in each video frame to obtain the tracking coordinates. These coordinates are used to isolate a region of interest for detecting the weld seam. This section is performed in two major steps, as shown in Figure 1: fingertip tracking and seam detection. Fingertip tracking uses a tracking-by-detection approach, whereas the seam detection process involves stereo image rectification, image distortion removal, region-of-interest isolation, edge detection, image patching, morphological operations, and removal of non-seam edges. All these methods were developed or improved in this paper.

#### 2.1.1. Fingertip Tracking

Fingertip tracking is performed by using a tracking-by-detection approach [7]. The tracking-by-detection approach is chosen because no occlusion occurs within the camera’s field of view during the demonstration, and the detection time from the trained deep learning network is approximately 70 ms. A live tracking process is performed, in which the demonstrator views rendered live footage of the hand poses in real time on a screen. The index finger is tracked to identify the region of interest (ROI) for seam line locations. The tracking coordinates form the image plane coordinates as

(1) $$T=\{\left(x_{i},y_{i}\right)|i\in \left\{1,\ldots ,n\right\}\},$$

where T is a set of image plane coordinates $\left(x_{i},y_{i}\right)$ for *n* hand tracking points.

A stereo image of the weldment is captured before the hand-tracking process begins. The seam detection process uses the hand-tracking coordinates in Equation (1) to isolate the ROI for potential seam detection. Once the seam region is isolated, the following image-processing operations are applied to detect the seam.

#### 2.1.2. Rectification and Distortion Removal

The captured image $I\in R^{m\times n\times 3}$ may contain lens distortion and must be undistorted [43,44] and rectified [45] to generate a rectified image $I_{r}\in R^{m\times n\times 3}$. Similarly, the hand tracking image plane points from Equation (1) must also be rectified to obtain $T_{r}$ such that $|T_{r}|=n$.

#### 2.1.3. Region of Interest

Finger tracking defines the region-of-interest coordinates in $T_{r}$ and is important because it isolates the seam line in the image. The first step in identifying the region of interest is to obtain a set of box coordinates B. Due to the discrete nature of the image, we select a scaling factor *f* in pixels and form a square box of size s=2f corresponding to each coordinate in $T_{r}$ such that

(2) $$B=\{\left(x_{i}-f,y_{i}-f,x_{i}+f,y_{i}+f\right)|i\in \left\{1,\ldots ,n\right\}\}.$$

To select the patch size, we first consider the hand box ratio $r_{b}$ from [8], which indicates the ratio of hand-to-image size. The hand box ratio ranges from $0.01<r_{b}<0.2$, with an average value equal to 0.16 [8], and is given as

(3) $$r_{b}=\frac{w\cdot h}{W\cdot H},$$

where (w,h) and (W,H) are the width and height of the hand bounding box and image, respectively. Assuming that the hand bounding box is a square, w=h. Since we are interested in the area around the fingertip, we consider a tenth of the bounding box length as s=h/10. Substituting w=h=10s and re-arranging the terms in Equation (3), we get

(4) $$s=\sqrt{\frac{W\cdot H\cdot r_{b}}{100}}.$$

Therefore, a scaling factor f=⌊s/2⌋ is used to develop image patches to isolate the region of interest, where the symbol ⌊·⌋ is used to round down the value.

The box coordinates in B for each tracking point are used to crop a patch of size (s×s). First, the rectified image $I_{r}\in R^{m\times n\times 3}$ is converted to a grayscale image $I_{g}\in R^{m\times n}$. A set of image patches *P* is then obtained by cropping the grey image $I_{g}$ with the box coordinates from Equation (2). These image patches are represented as

(5) $$I_{p}=\left\{\left(P_{i},\chi_{i}\right)|P_{i}\in R^{s\times s},\chi_{i}\in Z^{2},i\in \left\{1,\ldots ,n\right\}\right\},$$

where $I_{p}$ is a set of image patches $P_{i}$, and $\chi_{i}=\left(x_{i}-f,y_{i}-f\right)$ from Equation (2) are the top-left coordinates of the image patch in the image $I_{g}$. The top-left coordinates of the image patch are useful for patching the image.

#### 2.1.4. Edge Detection and Image Patching

Canny edge detection [32] is performed on each of the image patches in $I_{p}$ in Equation (5), similarly to [15,17]. The first step in patching these edge-detected image patches is to create an all-black coloured image $I_{b}\in R^{m\times n}$ such that

(6) $$I_{b}=\left\{b_{ij}=0|i\in \left\{1,\ldots ,m\right\},j\in \left\{1,\ldots ,n\right\}\right\},$$

where $b_{ij}$ is the *i*-th row and *j*-th column element of the matrix $I_{b}$, corresponding to *y* and *x* axes of the image coordinates, respectively. The patching of the image is done by using Algorithm 1. Once image patching is performed, the binary image $I_{b}$ will contain image patches that correspond to seam edges.
**Algorithm 1** Image patching algorithm takes the edge-detected image patches of size *s* in $I_{p}$ from Equation (5) and a black coloured image $I_{b}$ from Equation (6) and sequentially patches the image to construct a continuous edge line for the seam detection
 **Require:** 
$I_{p}$, the set of edge-detected image patches along with their top-left coordinates χ
 **Require:** 
$I_{b}$, the black-coloured single-channel image matrix initialised with all zero elements
 **Ensure:** 
$I_{b}$ reconstructed with contiguous edge segments1:  **for** $\left(P,\chi \right)\in I_{p}$ **do**2:        $y_{start}\leftarrow \chi \left(1\right)$                    ▹ Extract top-left row origin3:        $x_{start}\leftarrow \chi \left(0\right)$                  ▹ Extract top-left column origin4:        **for** *y*←1 **to** *s* **do**5:              **for** *x*←1 **to** *s* **do**6:                   $I_{b}\left(y_{start}+y-1,\;x_{start}+x-1\right)\leftarrow P\left(y,x\right)$7:              **end for**8:        **end for**9:  **end for**10:**return** $I_{b}$

#### 2.1.5. Morphological Operations

After edge detection and image patching, morphological operations are performed to ensure that the seam is connected. The first morphological operation performed on the image is dilation [46]. The purpose of the dilation operation is to expand the white pixels, thereby filling any gaps in the seam line. The dilation operation requires a kernel $K_{d}\in R^{p\times p}$ to perform the convolution operation over the image. Each element of the matrix $K_{d}$ is 1. The dilation function provides a new image $I_{d}\in R^{m\times n}$.

After the dilation operation, the image is filtered using a cross-correlation filtering [46] procedure with kernel $K_{f}\in R^{q\times q}$. The thinning operation [47] is performed after dilation and filtering to obtain a new binary image $I_{t}\in R^{m\times n}$ of a single pixel line for the detected edges.

#### 2.1.6. Removing Non-Seam Edges

The edges in the image $I_{t}$ may also contain some superfluous edges from scratches or other defects on the weldment. This could also be due to the line patterns of the welding table or the boundary edges of the weldment. Since our developed continuous weld seam search path is based on the seam edge, the other edge clusters must be removed. Therefore, an isolated cluster removal process must be performed to remove the smaller edges in the image that are not part of the weld seam.

For the binary edge-detected image $I_{t}$, there will be a set of pixels that defines the edges where the value of the pixel is 1. These foreground pixels form the set F of disjoint clusters $C_{i}$ such that $\left|F\right|=n_{k}$. These clusters are defined by the adjacency relations among pixel values, such as the 8-connectivity [46]. Each cluster $C_{i}\in F$ will contain the pixel coordinate that will not be part of any other cluster group such that $C_{i}\cap C_{j}=\emptyset$ for all i≠j.

The seam cluster selection process identifies the largest cluster. The largest size is defined by considering the area of the cluster $A(C_{i})$. The area of the cluster is computed by counting the number of pixels in each cluster. After calculating the area of each cluster, the cluster with the largest area is selected. The index *k* of the largest cluster is determined as

(7) $$k=\underset{i\in \{1,\ldots ,n_{k}\}}{\mathrm{argmax}}A\left(C_{i}\right).$$

The largest cluster $C_{k}\in F$ from the index *k* in Equation (7) is the set of seam coordinates S. The seam coordinates |S|=r are the set of *r* image coordinates used to find the continuous weld path for robot path following. For the continuous search, a search map can be created by first creating a black image $I_{s}\in R^{m\times n}$ where each element is 0, similar to Equation (6). The seam edge pixels for each pixel coordinate (u,v)∈S can be obtained by setting these pixel values in image $I_{s}\left(u,v\right)=1$.

### 2.2. Weld Seam Search Path

The continuous weld seam path is essential for the robot to perform the welding operation. A search algorithm is developed to find the continuous weld path. The first step in searching for the weld seam path is to determine the start and end coordinates of the seam. Once the start and end points of the seam edge are obtained, a path data structure is created to identify the continuous path. Finally, the data structure can be accessed to obtain a set of continuous coordinates.

#### 2.2.1. Seam End Points

The finger tracking coordinates $T_{r}$ can be used to determine the true start and end pixel coordinates of the seam. The approach assumes that the demonstrator starts and stops the demonstration slightly outside the true start and end locations of the weld seam, respectively. It is also assumed that the demonstrator does not move the finger back to the start location as an end position. The tracking coordinates do not need to be exactly on the weld seam, thus relaxing the constraints and permitting potential human errors due to tremor or human eyesight issues that can impact tracking. The start and end tracking coordinates can be obtained by using the first and last coordinates from $T_{r}$ as

(8) $$T_{S}=T_{r}\left(1\right)\;\mathrm{and}\;T_{E}=T_{r}\left(n\right),$$

where $T_{S}\in Z^{2}$ and $T_{E}\in Z^{2}$ are the start and the end rectified coordinates of fingertip tracking in the image coordinate plane, respectively.

The next step is to find the true start and end coordinates of the seam edge. The true start and end coordinates of the seam edges are the closest points to $T_{S}$ and $T_{E}$. Therefore, calculating the Euclidean distance between each of the seam coordinates $s_{i}\in S$ and Equation (8) for $T_{S}$ can be expressed as

(9) $$\Delta_{S}=\left\{\left(∥s_{i}-T_{s}∥\right)|s_{i}\in S,s_{i}\in Z^{2},i\in \left\{1,\ldots ,r\right\}\right\},$$

and similarly for $T_{E}$ as

(10) $$\Delta_{E}=\left\{\left(∥s_{i}-T_{e}∥\right)|s_{i}\in S,s_{i}\in Z^{2},i\in \left\{1,\ldots ,r\right\}\right\},$$

where $\Delta_{S}$ is a set of Euclidean distances between the tracking start and the coordinates in S, and $\Delta_{E}$ is a set of Euclidean distances between the tracking end and the coordinates in S. After calculating the Euclidean distances, the index of the shortest distance can be calculated as

(11) $$k_{S}=\underset{i\in \{1,\ldots ,r\}}{\mathrm{argmin}}\Delta_{s}\;\mathrm{and}\;k_{E}=\underset{i\in \{1,\ldots ,r\}}{\mathrm{argmin}}\Delta_{E},$$

where $k_{S}$ and $k_{E}$ are the indices of the shortest distance between the true start and end coordinates of the seam. Finally, the true start $\chi_{S}$ and end $\chi_{E}$ coordinates of the seam can be determined as

(12) $$\chi_{S}=s_{\left(k_{S}\right)}\;\mathrm{and}\;\chi_{E}=s_{\left(k_{E}\right)}.$$

#### 2.2.2. Search for Continuous Seam Path

After obtaining the start and the end coordinates of the seam edge from Equation (12), a search is performed using $\chi_{S}$ as the start point, $\chi_{E}$ as the goal point, and the binary seam edge image $I_{s}$ as the map where the white pixels with a value of 1 form the path.

Algorithm 2 is used to find the continuous path of the weld seam edge. This algorithm uses the start and end positions from Equation (12) and the binary image $I_{s}$. The algorithm uses a data structure called *Node* that contains two attributes: position, which indicates the pixel’s coordinate, and parent, which points to the previous *Node* in the path sequence.

In lines 1 and 2 of Algorithm 2, the Node($\chi_{S}$) and Node($\chi_{E}$) represents the start and end coordinates $\chi_{S},\chi_{E}\in Z^{2}$ given to the position attribute of *Node*. Line 3 records all the nodes visited by the search algorithm, while line 4 only maintains the record of the continuous final path, which is the output of the algorithm. Line 8 uses a 3×3 kernel *N* centred at the current pixel coordinate, and line 9 extracts the pixels with non-zero values to create a *kernel_path*. The *kernel_path* is an array of non-zero pixel coordinates within the confines of the kernel *N* created in line 8.

The function *sort_points_by_distance()* sorts the coordinates in the *kernel_path* in descending order of the Euclidean distance from the goal $\chi_{E}$, similar to Equation (9). Line 11 checks if *local_path* is a subset of *node_visited*, and if this condition is true, then the last node is removed from *path*. Lines 11 to 14 are an important and novel part of the search algorithm, as they backtrack the path to avoid getting stuck in the local spurs or edges.

From line 16 onwards, the coordinate positions χ in the *local_path* are assigned as the next node and the current node is assigned as the *next_node*’s parent. The algorithm returns an array *path* that consists of nodes from the *Node* data structure. The first node (the start point) in *path* will have no parent (the root node). Therefore, the next step will be to process the *path* array to obtain a set of continuous weld seam path coordinates.
**Algorithm 2** Seam search path algorithm takes the start $\chi_{S}$ and end $\chi_{E}$ coordinate positions along with binary seam edge image $I_{s}$ as a map to find the seam path
 **Require:** 
$\chi_{S}$, the start coordinate position of the seam edge
 **Require:** 
$\chi_{E}$, the end coordinate position of the seam edge
 **Require:** 
$I_{s}$, the binary seam edge image as the search map1:  start_node ← Node($\chi_{S}$)2:  goal_node ← Node($\chi_{E}$)3:  node_visited ← {start_node.position}4:  path ← {start_node}5:  current_node ← start_node6:  **while** current_node.position ≠ $\chi_{E}$ **do**7:        *x*, *y* ← current_node.position8:        N ← $I_{s}\left[y-1\ldots y+2,x-1\ldots x+2\right]$9:        kernel_path ← {(x−1+i,y−1+j)∣N(i,j)=1,∀i,j}10:      local_path ← sort_points_by_distance(kernel_path, $\chi_{E}$)11:      **if** local_path ⊂ node_visited = true **then**12:            remove_last(path)13:            current_node ← get_last(path)14:            **continue**15:      **end if**16:      **for** χ∈ local_path **do**17:            **if** χ∉ node_visited = true **then**18:                 next_node ← Node(χ)19:                 next_node.parent ← current_node20:                 node_visited.add(χ)21:                 path.add(next_node)22:            **end if**23:      **end for**24:      current_node ← next_node25:**end while**26:**return** path

#### 2.2.3. Path Coordinates Extraction

The *path* obtained using Algorithm 2 contains an array of data structures. This can be further processed by using another algorithm that traces the *Node* ancestry using its parent attribute.

Algorithm 3 provides a way to extract the coordinates of the *path* data structure. The process first uses the end coordinate value $\chi_{E}$ from Equation (12) and traces the parent *Node* until it reaches the start *Node* with position $\chi_{S}$.

Line 1 in Algorithm 3 uses the *path* and the goal $\chi_{E}$ to find the *Node* of the goal position. Then, the algorithm runs in a continuous loop from the goal node to keep tracing its parent node, and while doing so extracts the coordinate from the *position* attribute and adds it to the continuous seam path P such that

(13) $$P=\{\chi_{i}|\chi_{i}\in Z^{2},i\in \left\{1,\ldots ,n_{s}\right\}\},$$

where $\chi_{i}$ is the coordinates of the seam, and $\left|P\right|=n_{s}$ is the continuous set of $n_{s}$ seam  coordinates.

The continuous seam path coordinates from Equation (13) are useful for tracing the seam path from the start and finish points of the curve, as demonstrated by the human hand tracking. These path coordinates are still in the image plane of the rectified image. These points will be mapped in the robot coordinate space for the robot implementation. Therefore, with Equation (13), the seam path detection process is complete.
**Algorithm 3** Path ancestry tracing process that takes the array of *Node* data structure along with the goal position $\chi_{E}$ to return an array of path coordinates P from the start to the end of the seam edge
 **Require:** 
$\chi_{E}$, the end coordinate position of the seam edge
 **Require:** 
path, the array of *Node* indicating each coordinate position and its parent *Node*.1:  goal_node ← find_goal_in_path($\chi_{E}$, path)2:  current_node ← goal_node3:  P←∅4:  **while** true **do**5:        χ ← current_node.position6:        P.add(χ)7:        current_node ← current_node.parent8:        **if** current_node = NULL **then**9:              **break**10:      **end if**11:**end while**12:P.reverse()13:**return** P

### 2.3. Seam Detection Evaluation

Robot path following for the welding process requires a specified number of points to complete the operation. The minimum points required to perform welding on $n_{l}$ line segments is $n_{l}+1$. A single-line weld seam would require two points, whereas a saw tooth with three line segments would require four points. A curve can be broken into smaller $n_{l}$ line segments and will also require $n_{l}+1$ points. Considering the minimum number of $n_{l}$ points required to perform the welding operation, a set of user-defined ground truth checkpoints Γ for $n_{l}+1$ points is required such that

(14) $$\Gamma =\{\chi_{k}|\chi_{k}=\left(x_{k},y_{k}\right)\in Z^{2},k\in \left\{1,\ldots ,n_{l}+1\right\}\}.$$

The set of checkpoints mentioned in Equation (14) forms the ground truth for evaluation. The seam path developed in Equation (13) must pass through these checkpoints. Since there is a permissible tolerance of a few millimetres in the welding operation [16], a square bounding box is developed for each checkpoint of size b=2p+1, where *p* is the tolerance value in pixels. Given the user-defined ground-truth checkpoint Γ and a bounding box of size *b*, an algorithm is developed to determine the number of checkpoints visited by the seam path.

Algorithm 4 uses the continuous seam path P, the user-defined ground-truth checkpoints Γ, and the tolerance in pixel size *p*. In line 1, γ⊂Γ is a subset of all the checkpoints Γ, where the continuous seam path P passes through its bounding box. γ is initially set to the empty set and is populated as the seam path passes through the corresponding checkpoints.

Line 2 in Algorithm 4 uses a function *checkpoint_bounding_box()* that takes Γ and *p* as input. This function creates a set of bounding box $b=\left(x_{min},y_{min},x_{max},y_{max}\right)\in Z^{4}$ for each $\chi_{k}$ coordinate value in Γ as

(15) $$\beta =\{\left(x_{i}-p,y_{i}-p,x_{i}+p,y_{i}+p\right)|\chi_{i}=\left(x_{i},y_{i}\right)\in Z^{2},i\in \left\{1,\ldots ,n_{l}+1\right\}\}.$$

Line 5 in Algorithm 4 uses a function *checkpoint_pass_check()* that takes $\chi_{p}\in P$ and b∈β and returns a boolean *true* if the coordinates $\chi_{p}=\left(x,y\right)$ are within the area defined by bounding box $b=(x_{min},y_{min},x_{max},y_{max})$ by performing logical operation

(16) $$\left(\left(x_{min}<x\right)\wedge \left(x<x_{max}\right)\right)\wedge \left(\left(y_{min}<y\right)\wedge \left(y<y_{max}\right)\right),$$

where ∧ is the symbol for the logical **AND** operation.

In line 6 of Algorithm 4, if the seam path passes through one of the checkpoints, then the checkpoint is added to the set of visited checkpoints. In line 7, the index of the coordinate $\chi_{k}\in \Gamma$ is assigned to the index variable (idx). In lines 8 and 9, the values associated with the index (idx) in β and Γ for bounding boxes and ground truth checkpoints, respectively, are removed. Finally, the algorithm returns the set of checkpoints passed by the continuous seam path P.

The set of passed checkpoints $\gamma_{p}$ obtained as the output of Algorithm 4 and the total number of user-defined ground truth checkpoints Γ can be used to calculate a passing accuracy as

(17) $$r_{p}=\frac{\left|\gamma_{p}\right|}{\left|\Gamma \right|},$$

where $r_{p}$ is the passing accuracy, $\left|\gamma_{p}\right|$ is the number of passed checkpoints, and |Γ| is the total number ground truth checkpoint. The suffix *p* indicates the tolerance in pixels such that $r_{0}$, $r_{1}$, and $r_{2}$ represent the passing accuracy of checkpoints for 0, ±1, and ±2 pixels, respectively. Using the passing accuracy $r_{p}$, the detection accuracy can be evaluated. Furthermore, the tolerance value *p* can be adjusted according to the strictness of the evaluation criteria.
**Algorithm 4** Checkpoint seam detection evaluation algorithm that takes a set of continuous weld seam path coordinates P, a set of user-defined ground truth in the form of checkpoints Γ, and the tolerance in pixel size *p*
 **Require:** 
P, a set of continuous weld seam path coordinates
 **Require:** 
Γ, a set of user-defined ground truth checkpoint coordinates of size $n_{l}+1$
 **Require:** 
*p*, tolerance in pixel size to create a bounding box over checkpoints1:  $\gamma_{p}\leftarrow \emptyset$2:  β← checkpoint_bounding_box(Γ, *p*)3:  **for** $\chi_{p}\in P$ **do**4:        **for** $b\in \beta ,\chi_{k}\in \Gamma$ **do**5:              **if** checkpoint_pass_check($\chi_{p}$, *b*) = true **then**6:                   $\gamma_{p}$.add($\chi_{k}$)7:                   idx←Γ.index($\chi_{k}$)8:                   β.remove(idx)9:                   Γ.remove(idx)10:                 **break**11:            **end if**12:      **end for**13:**end for**14:**return** $\gamma_{p}$

### 2.4. Composite Weld Seam Detection

The hand-tracking-to-weld-seam detection process can be incorporated to detect composite weld seams. The composite weld seams are defined as weldments with multiple weld lines or curves. The process for composite weld seam detection is similar to that for single-weld seam detection, as shown so far in this section. Therefore, the process remains the same for multiple-line detection, with the addition of multiple hand tracking.

The hand tracking will be performed on multiple seam lines and stored as

(18) $$T_{c}=\left\{T_{i}|i\in \left\{1,\ldots ,n\right\}\right\},$$

where $|T_{c}|=n$ is a set of the set of hand tracking points $T_{i}$ for *n* weld seams. Each one of the $T_{i}$ represents the tracking coordinates, as shown in Equation (1). The seam path coordinates can be obtained for the composite weld seams by following the methods developed in this research for individual seam detection. These seams can be merged to obtain a composite weld seam.

### 2.5. Robot Seam Path Implementation

**Figure 2:**
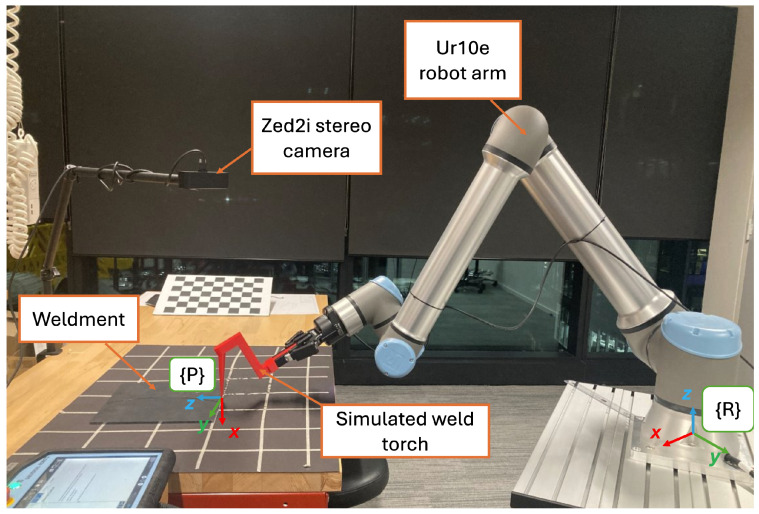
Experimental setup including the robot, 3D-printed simulated weld torch, weldment, and Zed2i stereo camera.

The continuous path coordinates for the seam P obtained from Equation (13) are in the image coordinate space. These coordinates must be transformed into the robot’s coordinate frame. The weld path implementation requires a fixed robot coordinate frame {R} and another coordinate frame at the tip of the end-effector (welding torch) {P}, as shown in Figure 2. To avoid “welding from underneath”, the welding table was set as the constraint for the “floor”. This “floor constraint” prevents the robot from approaching the weld path from underneath the weld table.

The continuous weld seam path P consists of a set of $\left(u,v\right)\in Z^{2}$ seam pixel coordinates in the image plane. The continous seam path coordinates P in the image plane are transformed to the robot space coordinates R, consisting of $\left(x_{r},y_{r},z_{r}\right)\in R^{3}$ robot space coordinates by performing stereo camera and hand-to-eye calibration presented in previous work [24] to obtain camera re-projection matrix *Q* and camera to robot transformation matrix TCR.

The continuous weld seam path coordinates (u,v) along with the corresponding disparity values, obtained from the stereo disparity map D(u,v)=d for depth extraction, can be used to form a vector $v={\left(\begin{array}{cccc} u & vs. & d & 1 \end{array}\right)}^{T}$. The camera coordinates are obtained as

(19) $$\tilde{x}=Q\cdot v,$$

where $\tilde{x}={\left(\begin{array}{cccc} \tilde{x} & \tilde{y} & \tilde{z} & \tilde{w} \end{array}\right)}^{T}$, and $x_{c}=\tilde{x}/\tilde{w}={\left(\begin{array}{cccc} x_{c} & y_{c} & z_{c} & 1 \end{array}\right)}^{T}$ is the camera frame coordinate of the respective continuous path coordinate in P. The point $x_{c}$ can be projected in the robot’s coordinate frame as

(20) $$x_{r}{=}^{R}T_{C}\cdot x_{c},$$

where $x_{r}={\left(\begin{array}{cccc} x_{r} & y_{r} & z_{r} & 1 \end{array}\right)}^{T}\in R$ is the equivalent continuous path coordinate in the robot’s coordinate frame.

The continuous path coordinates of the torch can be obtained as

(21) $$P_{P}{=}^{P}T_{R}\cdot P_{R},$$

where $P_{R}\in R$ is a welding path point in the robot coordinate space {R}, $P_{P}$ is equivalent welding point in frame {P}, and TRP is the transformation matrix from robot {R} to {P} obtained using [48]. Once $P_{P}$ is obtained, the robot can be controlled to move to the point for welding.

## 3. Results

In this section, we present the experimental setup, the computers, and the software used in this research to validate the methods. First, we present the results on hand tracking, which builds on previous hand-pose detection work [8]. A step-by-step process, including intermediate results in the form of images, is presented for straight-line seam detection based on the methods presented in Section 2.1. The final results for other seam types, along with the search path implementation, are also presented and follow the same steps as the straight-line weld seam. Furthermore, we present composite weld path detection results. Finally, the seam path is implemented on the UR10e robot, and we provide visual inspection of the path in both the ROS2 RViz2 visualisation software and the camera frames of the video of the robot executing the weld seam path.

### 3.1. Experimental Setup

The experimental setup comprises a UR10e robot from Universal Robots A/S (DK-5260 Odense S, Denmark), a ZED2i stereo camera from StereoLabs (San Francisco, CA, USA), a 3D-printed simulated weld tool, and a weldment piece. The experimental setup is shown in Figure 2. The setup, along with the simulated weld torch (identified as the pointer tool in [24]) schematics, is the same as presented in previous work [24].

### 3.2. Computer and Software

The computational and software specifications in this work are similar to those in previous work [24]. The hand detection and tracking development work was performed in Python 3.10.12 using PyTorch 2.2.1+cpu [49], a deep learning framework. Computer vision tasks were performed using OpenCV [50]. ROS2 Humble (Robot Operating System) [51] was used for robot path planning.

### 3.3. Hand Tracking Results

The previous work outlines the development and testing of the hand-pose detection process [8]. In this work, a tracking-by-detection approach is used to detect hand pose in each frame using a trained deep network. Real-time detection is performed, and only the coordinates of the tip of the index finger are stored, as per Equation (1).

**Figure 3:**
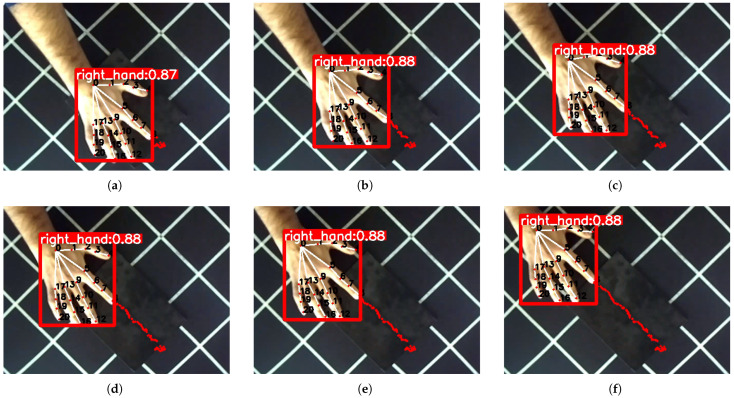
Hand tracking performed using a tracking-by-detection approach and a trained hand-pose YOLOv8 pose network. (**a**–**f**) Screenshots of hand-pose detection and tracking the tip of the index finger at incremental time points.

The tracking process is shown in Figure 3. The tracking process begins when the hand appears within the camera’s field of view. The demonstration needs to be performed from the start to the end of the weld seam. A detection confidence threshold of 0.8 is set, meaning that tracking points are recorded only when the hand detection confidence exceeds this threshold.

The time required to detect a hand in each frame is 70 ms using the trained hand-pose network [8]. The OpenCV video capture function is set to read 10 frames per second. The detection performed using tracking-by-detection has enough time to process each frame individually. Moreover, real-time detection and rendering enable the user to observe the tracking process on a monitor. This process ensures that only the tracking coordinates are stored, not the video. Since the video files are not stored, there is no need for a large-scale data storage system.

### 3.4. Seam Detection Results

The seam detection process begins after collecting the hand-tracking points and rectifying them. A stereo image, each of size (672×376×3) of the weldment, is captured. This image is converted to grayscale, resulting in a single-channel image of size (672×376).

**Figure 4:**
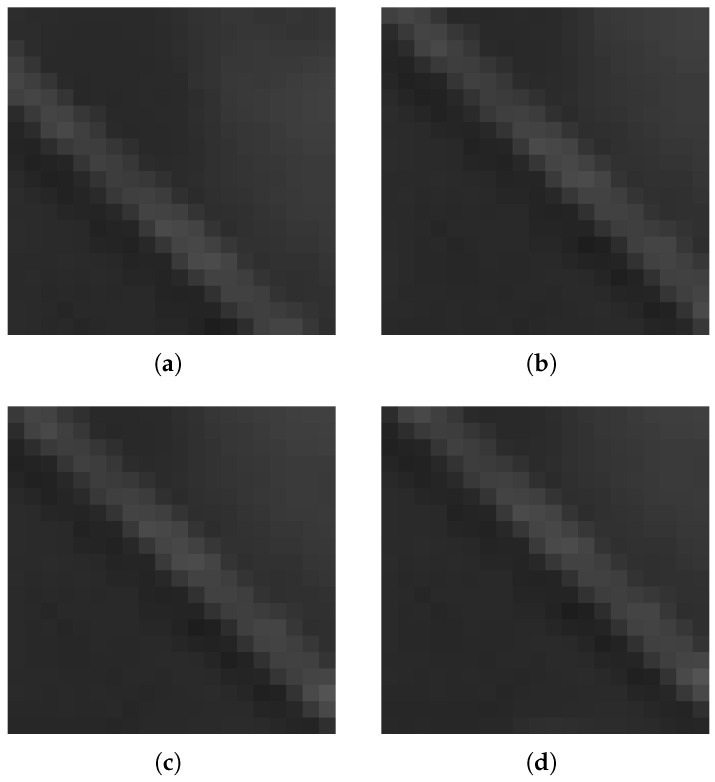
Extracting image patches using the tracking point coordinates. (**a**–**d**): Sample patches from the bounding box extraction of the hand tracking coordinates.

**Figure 5:**
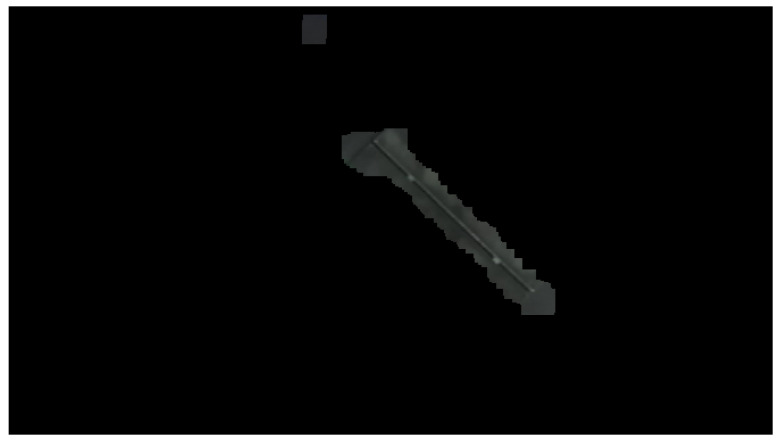
Region of interest over the weld seam line with each of the patches.

The seam detection process begins by extracting a small image patch of size (s×s), as shown in Equation (5). Substituting $r_{b}=0.16$ from [8] and the size of the image (W,H)=(672,376) in Equation (4), we get s≈20. Therefore, a scaling factor f=⌊s/2⌋=10 and a patch of size (20×20) is used. Figure 4 shows the image patches of size (20×20) represented in Equation (5). These patches can be visualised over the entire weld seam in Figure 5.

**Figure 6:**
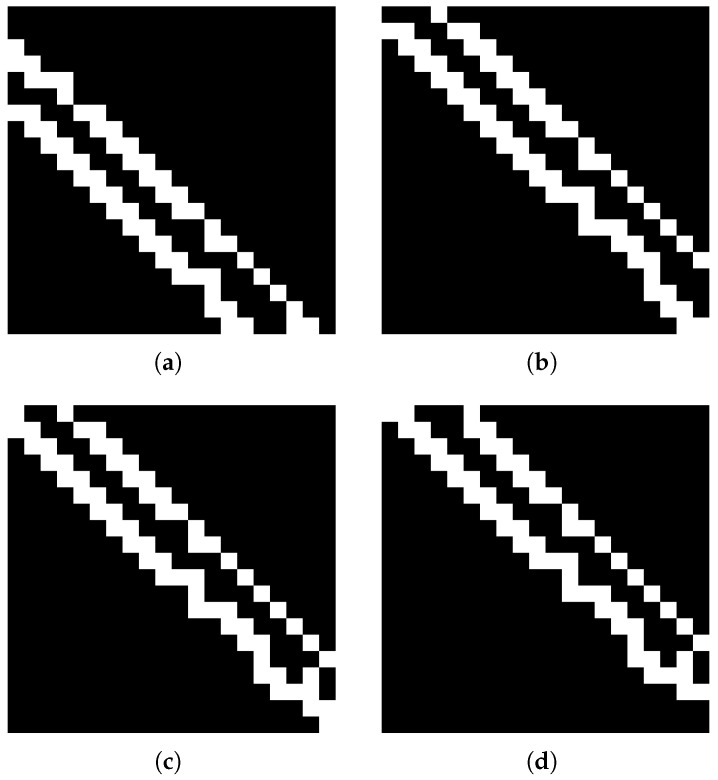
Edge detection on each of the patches. (**a**–**d**): Edge-detected sample patches from the bounding box extraction of the hand tracking coordinates.

**Figure 7:**
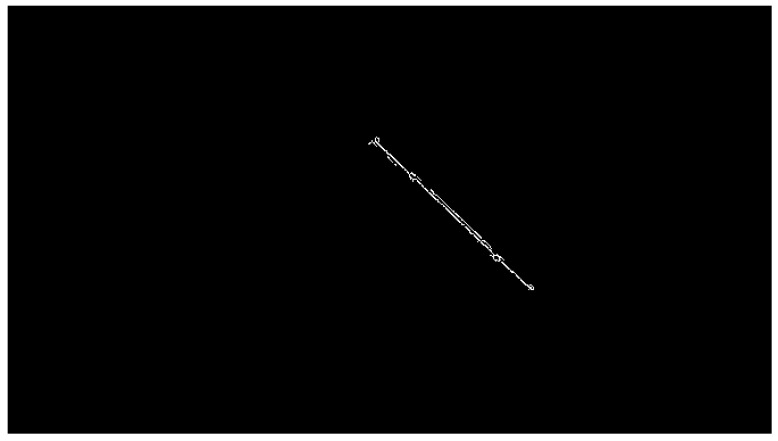
Patching all the small patches to obtain the seam edge.

Canny edge detection [32] is applied to each image patch to obtain the seam edges, similarly to [15,17]. Figure 6 shows a sample of the Canny edge detection performed on the same patches of images from Figure 4. Once edge detection is performed on each image patch, the patches are reassembled into the image using Algorithm 1. Figure 7 shows the result of patching the image using Algorithm 1.

**Figure 8:**
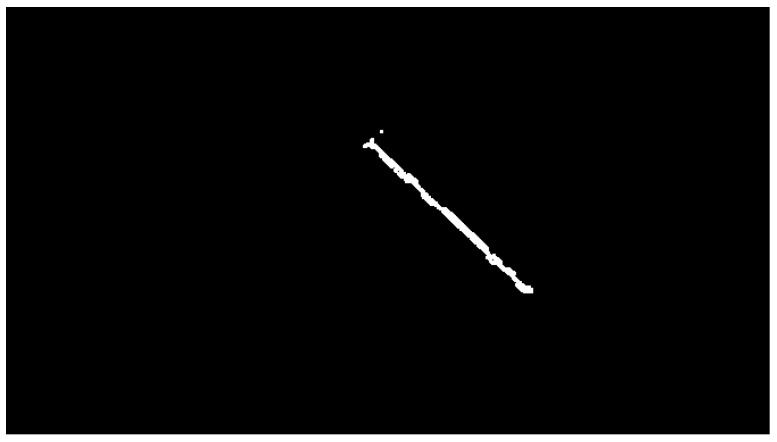
Image dilation connects all the broken edges, giving one coherent edge body.

**Figure 9:**
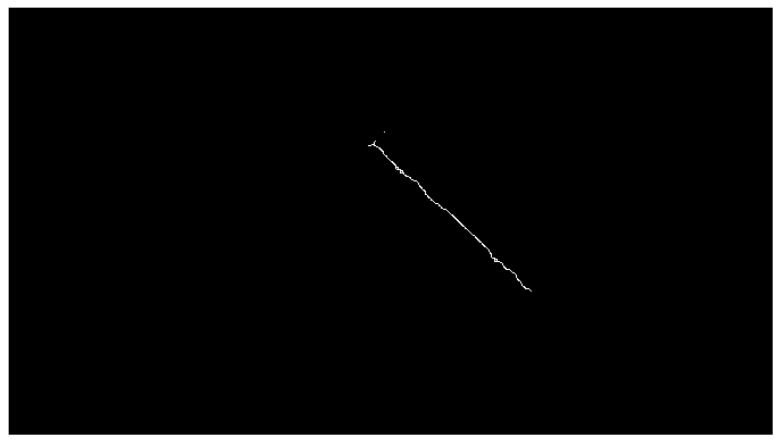
Thinned image provides an edge of single pixel thickness.

The image dilation is applied to the image in Figure 7 to fill gaps and reconnect broken edges. In this research, a dilation kernel of size (3×3) is selected. Figure 8 shows the dilated image. Finally, an averaging filter with kernel size (5×5) is performed on the dilated image, followed by a thinning operation. The above-mentioned kernel sizes and filtering process are selected from the recommendations in [52]. Figure 9 shows the result of the thinning operation. This image will be used to extract the non-zero pixels for the seam image in Algorithm 2.

**Figure 10:**
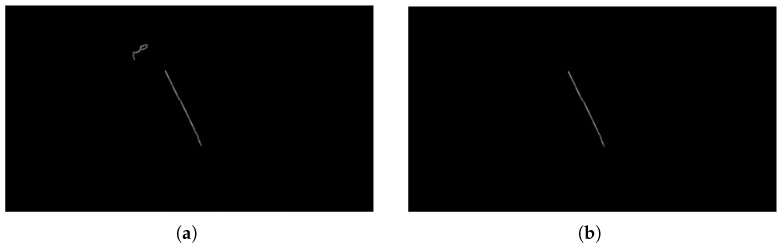
Removing a disjoint cluster to maintain a single coherent and continuous seam edge. (**a**): Edge detection with a small disjoint cluster. (**b**): Disjoint small cluster removed and seam edge maintained.

In some cases, smaller edges that are not part of the seam may be detected in the image. These edges result from the hand appearing over the edges of the workbench when it first enters the camera’s field of view. For seam path search, only one coherent and continuous seam edge is required. Therefore, the superfluous edges are removed. Figure 10a shows the presence of the disjoint cluster, and Figure 10b shows that the disjoint cluster is removed and only the seam edge is maintained.

### 3.5. Search Path Results

The search path process uses the image in Figure 9 and its non-zero coordinates to determine the seam path. The methods and algorithms presented in Section 2.2 are used to find the continuous search path. Experiments are performed to detect weld seams in three types: line, curve, and saw tooth. Visual evaluation is performed for each one of these weld seams.

#### 3.5.1. Checkpoint Evaluation Results

The quantitative results presented in this section are obtained using the checkpoint evaluation method described in Section 2.3. The experiments were performed by placing the weldment at various locations and orientations on the workbench under different lighting conditions in the room.

**Figure 11:**
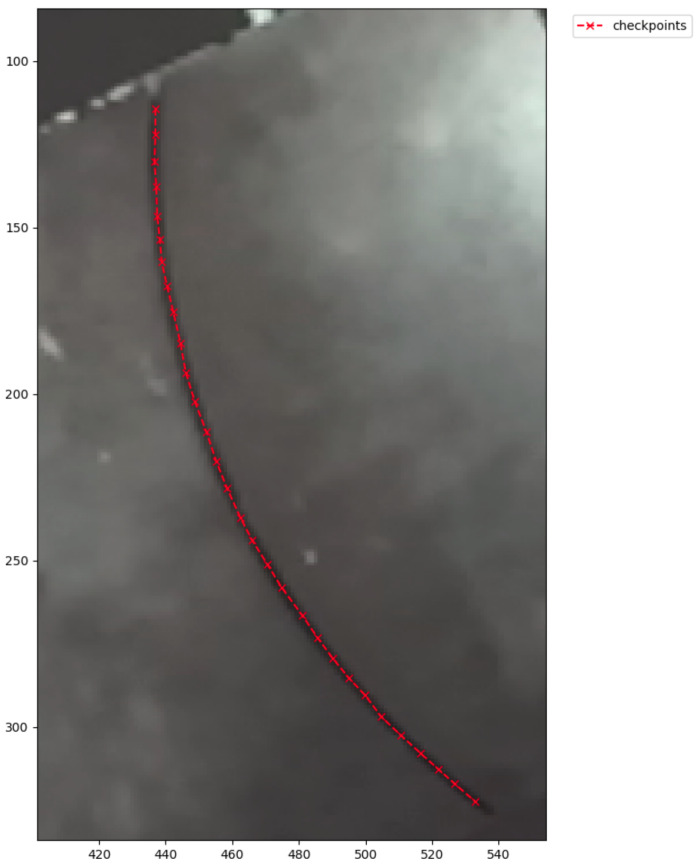
Software tool developed in Python that enables the user to save the checkpoint coordinates in the image plane.

A software tool was developed in Python to allow the user to click on an image to define checkpoints. Figure 11 shows the user interface of the developed tool, which saves the checkpoint coordinates in the image plane of the rectified image. These coordinates are used as the checkpoints for evaluation.

The quantitative results for line, curve, and saw tooth seam in different positions and orientations are presented in Table 2. The results presented in Table 2 consists of number of user-defined total checkpoints |Γ|, number of passed checkpoints $\left|\gamma_{p}\right|$, accuracy measured as passing ratio $r_{p}$ from Equation (17), and error $\epsilon_{p}=1-r_{p}$. A 0 pixel tolerance indicates an exact match for the seam coordinates and the checkpoints. Using ±1 and ±2 pixels provides a small tolerance. In the setting of our experiment, a 1 pixel accuracy is equivalent to a 1 mm tolerance in robot space. This is an acceptable limit for welding operation [16].

**Figure 12:**
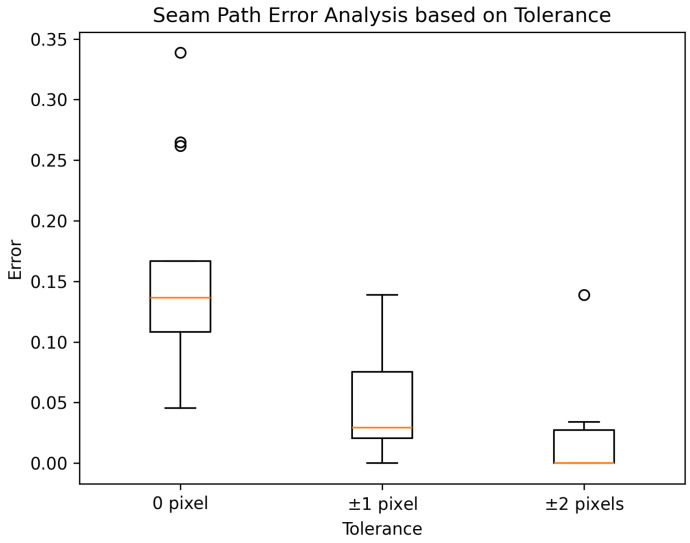
Error plot for checkpoint with tolerance of 0 pixels and ±1 pixels.

Figure 12 shows the error plots in terms of checkpoint passing error from Table 2 for 0, ±1, and ±2 pixels. For a 0 pixel tolerance, the median error is 0.13 with three outliers having higher error due to stricter tolerance criteria. For a ±1 pixel tolerance, the median error is 0.029 and for ±2, the median error is 0. This means that the median accuracy over different types of seam paths is 0.971 (>97%) for ±1 pixel tolerance. In addition to that, the overall mean accuracy for ±1 pixel tolerance from all the seam type experiments is 0.957 (>95%). Figure 12 also shows how the median error drops with an increment in the pixel tolerance criteria.

#### 3.5.2. Search Path Evaluation Results

**Figure 13:**
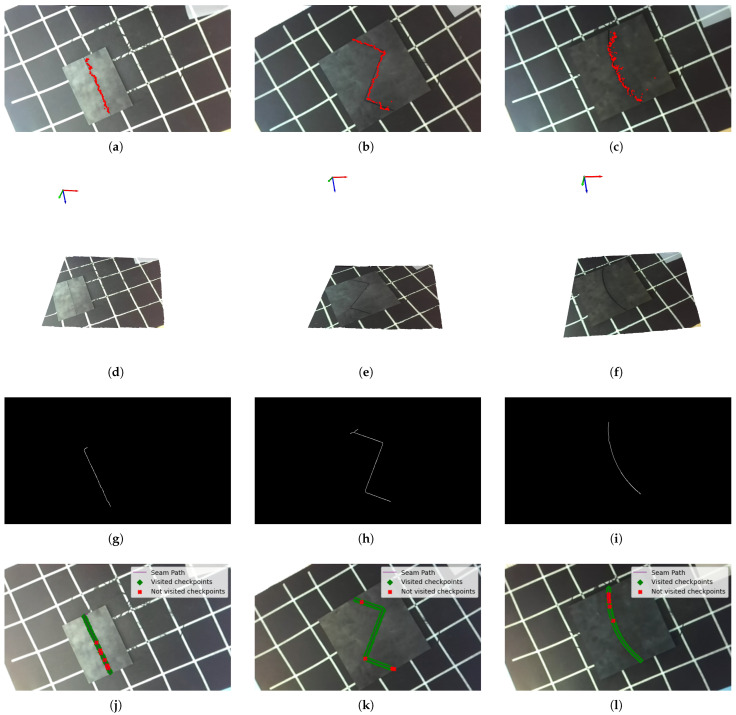
Qualitative evaluation of the weld seam detection. (**a**–**c**): Rectified left image of the stereo camera with hand tracking coordinates for the tip of the index finger. (**d**–**f**): Point cloud visualisation of the weldment using a stereo camera. (**g**–**i**): Seam detection. (**j**–**l**): Checkpoint evaluation for 0 pixel tolerance.

Qualitative analysis is performed to evaluate seam detection visually. The results for qualitative analysis are presented in Figure 13. These results outline the entire seam-detection process, starting with capturing stereo images, performing a hand-tracking demonstration, estimating depth, detecting the seam line, and searching for the seam weld path.

Figure 13a–c show the rectified left image from the stereo camera for line, saw tooth, and curve weld seams, respectively. Hand tracking is performed for each weld seam, and the hand-tracking coordinates, along with the stereo images, are saved. These images and coordinates are then rectified.

Figure 13d–f show the point cloud generated by stereo matching. The camera frame axes are also visible in the point cloud images. Each point in the point cloud is expressed in the camera reference frame. Initial viewing of the point clouds can also help determine whether a direct light source is causing surface reflection on the weldment.

Figure 13g–i show the seam detection as a single-pixel-wide edge for line, saw tooth and curve, respectively. Although these edges represent the seam type distinctly, they cannot provide a continuous weld path for the robot to follow. The continuous weld path is implemented in Figure 13j–l. These continuous path coordinates lie in the rectified image plane, and their corresponding points can be found in camera coordinates and eventually projected into robot coordinate space. Figure 13j–l also show the visual checkpoint evaluation results. The purple line represents the continuous seam path, the green marker represents the passed checkpoints, and the red cross represents the missed checkpoint for 0 pixel tolerance.

**Figure 14:**
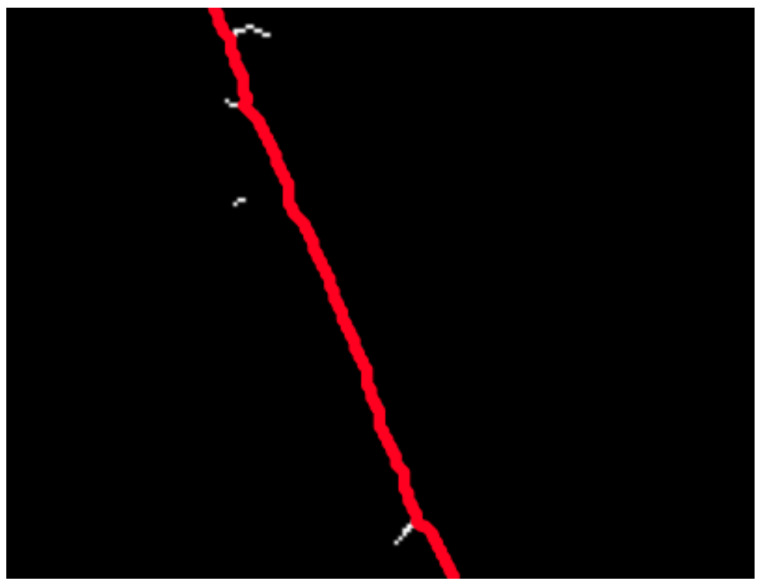
Evaluation of the seam path on local branches and spurs. Red pixels indicate the continuous weld seam path, and white pixels are local branches and spurs. The image is magnified to focus on local branches and spurs.

The backtracking process of Algorithm 2 is an important capability that avoids local branches and spurs on the weld seam during the thinning process. Figure 14 shows a magnified segment of the image with different spurs and branches appearing on the weld seam during the thinning process. The spurs and local branches are white pixels, and the red-coloured pixels represent the continuous weld path in Figure 14. These local branches are not the endpoint of the seam as per the user demonstration. Therefore, if the algorithm gets stuck in these spurs or local branches, it backtracks and avoids those branches when developing a continuous weld seam path.

#### 3.5.3. Search Algorithm Analysis

The seam path search was implemented using Algorithm 2. Dinham and Fang implemented a similar weld seam search [16] using the A* search algorithm. While the A* search algorithm [35] is widely used as a general-purpose search algorithm, its computation of heuristic cost for each available node may not be ideal. Due to the initial image pre-processing steps, a simple start-to-end search algorithm with backtracking capability, as highlighted in Algorithm 2, is proposed as an alternative.

In this section, we analysed the performance of the A* search algorithm and Algorithm 2. We considered measuring the time and memory utilisation of both algorithms. Since the search path also involves visiting all the available places called *Nodes*, we measured how many nodes the algorithms generate, which impacts the memory utilisation. Table 3 shows the analysis performed on the data collected on line, curve, and saw tooth weld seam types. In Table 3, *Visited Nodes* is the number of available space the algorithm visits before finding the final path, *Seam Nodes* is the number of seam coordinates that indicate the path, and ΔNodes=(VisitedNodes−SeamNodes) is the difference between the number of visited nodes and the number of actual seam path coordinates. Time is measured in seconds, and peak memory utilisation is measured in Megabytes (MB).

To ensure fairness in testing A*, instead of developing it ourselves, we used an open-source algorithm developed in the Python programming language [53]. This open-source program can only travel up, down, left, and right. Since our seam path is a single-pixel wide, the open-source program would not function. Thus, we added the capability to move diagonally. These modifications to the open-source A* search algorithm do not reflect the algorithm’s inability, but the implementation of the software program. After the necessary modifications to the open-source program, we used the seam path data to test the output.

The results of testing both A* and Algorithm 2 are presented in Table 3. The results show that the total number of nodes evaluated by A* search is more than that of Algorithm 2. A* search evaluates the heuristic cost for the nodes that form small branches and spurs in the seam path. Algorithm 2 avoids checking each path and only backtracks when it reaches dead ends on small branches and spurs. This visit of a node is observed in memory utilisation, where the mean of the memory used for A* search is 6.72 MB, while that for Algorithm 2 is 4.7 MB. These spikes in memory usage were tested by using the *malloc* module in Python. Both algorithms take tens of milliseconds to find the respective seam path. Therefore, while A* is a general-purpose search algorithm, using Algorithm 2 as a special-purpose algorithm that does not check the movement cost for each node for the weld seam is a useful alternative.

### 3.6. Composite Path Evaluation

**Figure 15:**
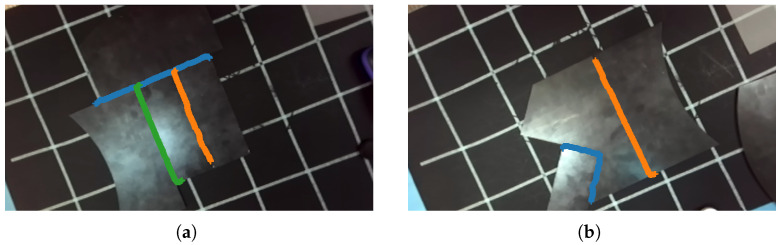
Composite seam path detection. (**a**): Three seam path detections on composite weldments. (**b**): Two seam path detections on composite weldments.

Composite seam path detection follows a similar process: hand tracking is performed on each seam, and each set of tracking data is processed to detect the path. The methods to detect the composite path are mentioned in Section 2.4. Figure 15 shows the experiments performed on composite weldments. The different seam paths are rendered in different colours to differentiate the segments. Figure 15a consists of three seam paths and Figure 15b consists of two seam paths. Each tracking is processed individually, and the paths are then implemented in segments.

### 3.7. Robot Path Implementation Results

The robot path implementation uses the image-plane continuous path coordinates, maps them to camera coordinate space, and finally to robot coordinate space. Once these seam coordinates in the robot space are available, they are sent to ROS2 MoveIt2 [54], a ROS2 software tool for robot path planning. First, the path planning is visualised in RViz2, a ROS2 visualisation tool, and then executed on the actual robot.

**Figure 16:**
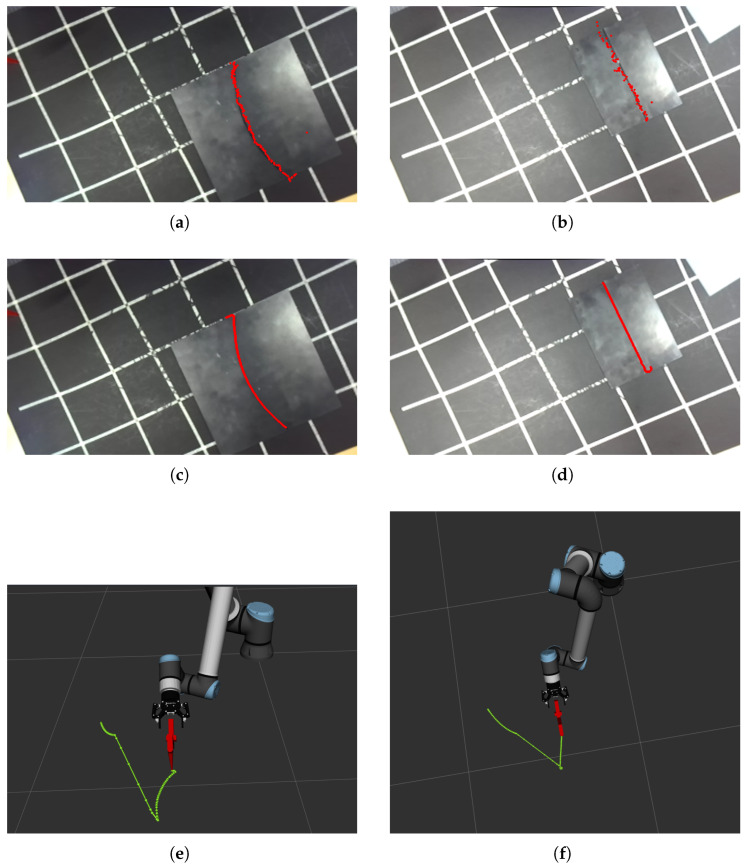
Robot path implementation includes hand tracking over the seam, detecting the seam and continuous path, and implementing path following on the robot. (**a**,**b**): Hand tracking on weld seam. (**c**,**d**): Continuous path detection and checkpoint evaluation. (**e**,**f**): ROS2 implementation of the robot path following.

Figure 16 shows the complete process from detecting hand tracking coordinates in the image plane to executing the weld seam path in the robot coordinate space. Figure 16a,b show the curve and line seam, respectively, along with the finger tracking coordinates. Figure 16c,d show the continuous seam path implementation in the camera frame. Figure 16e,f show the robot path planning and implementation process in ROS2 for the respective paths.

**Figure 17:**
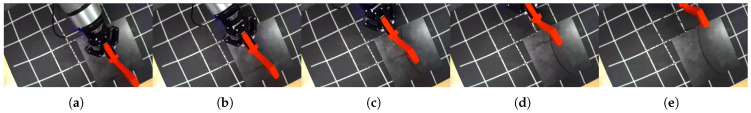
Video frames of the robot path implementation for the curve seam. (**a**–**e**): Video frames of the robot position at different time intervals.

**Figure 18:**
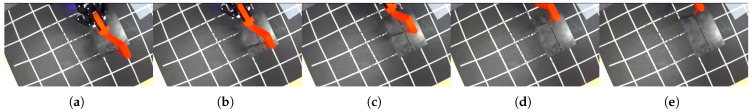
Video frames of the robot path implementation for a straight line seam. (**a**–**e**): Video frames of the robot position at different time intervals.

Figure 17 and Figure 18 show screenshots of the video recording of the robot seam path implementation for curve and straight line seam types, respectively. These frames correspond to their respective weld seam types in Figure 16. The robot speed is set to 20% of the maximum velocity of the UR10e robot on the teach pendant controller. This speed can be adjusted as needed for the welding process.

The camera in the experimental setup is placed approximately 500 mm from the workbench. The RMS error reported in the hand-to-eye calibration process in previous work [24] are $x_{\mathrm{RMSE}}=0.8469$ mm, $y_{\mathrm{RMSE}}=0.6713$ mm, and $z_{\mathrm{RMSE}}=1.7402$ mm in the x, y, and z direction, respectively. The same robot hand-to-eye transformation matrices were used to perform the hand-to-eye calibration.

Using the hand-to-eye calibration process [8], the values of the camera re-projection matrix and camera-to-robot transformation matrix given in Equations (19) and (20) are obtained as

Q=100−312.04010−186.02000472.33008.3070andTCR=0.8320.548−0.02070.9430.553−0.836−0.0262−0.519−0.0310.0103−0.9990.5030001.

We select a group of random adjacent pixel coordinates, where the adjacent pixel coordinates in a group are 1 pixel apart. We project these image coordinates to robot coordinate space using Equations (19) and (20). For two adjacent pixel coordinates in the image plane and their stereo disparity values, the Euclidean distance between these two points can be calculated. Table 4 shows the group of two adjacent pixel coordinates (u,v), their stereo disparity value *d*, and equivalent robot coordinates $\left(x_{r},y_{r},z_{r}\right)$, with (Δx,Δy) as the distance between each point in the *x* and *y* directions in the robot coordinate space, and the Euclidean distance in robot space calculated as $\sqrt{{\Delta x}^{2}+{\Delta y}^{2}}$.

**Figure 19:**
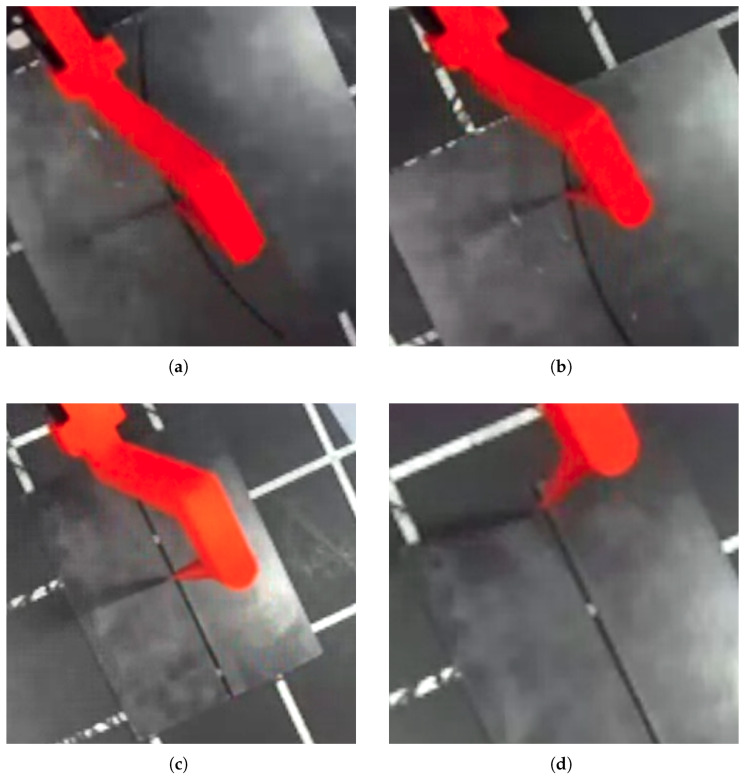
Magnified images of the robot path implementation. (**a**): Magnified image of Figure 17c. (**b**): Magnified image of Figure 17d. (**c**): Magnified image of Figure 18c. (**d**): Magnified image of Figure 18e.

In Table 4, Groups 1–4 have a 1-pixel distance between the adjacent pixel coordinates. Group 5 is used to evaluate the mapping of a 2-pixel distance from the image to robot coordinate space. From Table 4, for Groups 1–4, the mean euclidean distance is 0.00104 m (≈1 mm). For Group 5, when the pixel distance was 2 pixels, the equivalent robot space distance was 0.00207 m (≈2 mm). The robot seam path implementation can be further evaluated by visual inspection over the weld seam. Figure 19 shows a magnified image of the simulated weld torch from Figure 17 and Figure 18, where the simulated torch follows the seam path.

## 4. Discussion and Limitations

In this section, we discuss the methods, evaluation process, and results. We also compare the approach with other methods and highlight its strengths and limitations.

### 4.1. Seam Detection Process

The seam detection process is based on image processing methods. The image processing approach has been developed to accommodate different use cases, including seam curve types and environmental conditions. The different types of seam curves selected in this research form the basis for various weld seams. It can be observed that the saw tooth seam is composed of smaller line segments. Similarly, curve segments can also be considered an integration of small line segments. Therefore, the advantages of the methods developed here are that they can generalise to other types of seam curves.

The continuous weld seam path search algorithm developed in this research runs in less than a second. This time is negligible and very similar to that of using Probabilistic Hough Transforms [55]. An additional advantage of the search algorithm developed in this research is that, unlike Hough Transforms, it is not limited to straight lines [56]. Furthermore, determining additional path coordinates can be beneficial, as it helps develop smooth robot path planning. Furthermore, the seam is a single-pixel wide, which means that performing morphological operations provides additional benefits to the search process. Unlike algorithms such as A* search used for weld search paths [16], the search algorithm in this research does not require computing the heuristic cost as a function of Euclidean or Manhattan distance. Therefore, the search path developed in this paper is a useful alternative to A* for continuous weld seam paths.

The limitations of the search method are that it records every consequent pixel value. Saturation of pixel values within regions along the path can cause jerky motion in the robot weld path implementation. This problem can be overcome by omitting a few coordinates, which allow the robot path planner to extrapolate smooth motion. Future work involving comparisons with graph traversal-based search algorithms can provide additional insights into developing a continuous seam path for robotic welding. Furthermore, a filtering process can be developed to ensure smooth trajectories.

### 4.2. Evaluation Process

In this research, we evaluated seam detection using qualitative analysis by rendering the detected weld path on the image. The analysis of the rendered result is performed by observation to ensure a tolerance of ±1 pixel. The rendered images with the weld path superimposed are essential for evaluating the detection process. Mapping 1 pixel in the image plane to its equivalent distance in physical space also helps measure welding accuracy. Passive seam detection methods such as [16] show an acceptable error of ±1 mm. In this paper, we also evaluated that more than 95% of the points tracked are within ±1 mm.

Most modern robot controllers use different types of path planners, such as MoveL, MoveJ, and MoveC, which correspond to linear, joint, and circular motion, respectively. A line segment requires two user-specified points to implement MoveL, and a curve requires at least three points for circular path planning. However, a curved path can also be implemented using the MoveL command if the curve is treated as a segment of smaller connected lines. Therefore, the continuous weld path coordinates obtained using the method presented in this research can be fed to any robot controller as small line segments for the MoveL function, which can still generalise the path implementation.

### 4.3. Environmental Conditions

Most of the weldment pieces have reflective surfaces. A direct light source on top of the weldment is not ideal for seam detection with cameras. Furthermore, a dark environment is not ideal for hand tracking. Therefore, it is essential to consider the environmental conditions while setting up the robot.

In this research, the experiments were conducted in ambient daylight. This provides sufficient illumination for hand tracking to detect the hand while avoiding reflectance from the weldment. However, when the experiments were performed with the overhead light, hand tracking and seam detection still worked, but depth estimation over the seam was affected. This problem is overcome by ensuring that the overhead light is not reflected from the seam on the weldment by repositioning the weldment before tracking. Using point clouds from stereo images is a helpful tool for evaluating whether external light can affect depth estimation.

### 4.4. Human–Robot Interaction Robustness

There are several parameters to consider in human–robot interaction (HRI). Human errors in demonstrations can lead to potential failures in robot task implementation. These human errors can be in the form of physiological conditions such as tremors. Other potential errors could be due to suboptimal demonstration. Furthermore, environmental factors can also affect human demonstrations, such as occlusions and specular reflections. Therefore, addressing these problems is essential to highlight the robustness and limitations of the system.

This research incorporates different strategies that can address some of the failure modes. For example, by not requiring the hand movement to be superimposed on the weld seam, potential human hand tremor issues can be addressed. The methods developed in this research use a YOLO-based hand-tracking-by-detection approach, since YOLO has demonstrated real-time application. The live rendering of the hand pose along with the tracking points, as shown in Figure 3, assists the demonstrator in ensuring the demonstration is done correctly. Regarding environmental error in HRI, the welding workbench is kept free of occlusion. Furthermore, the hand detection network [8] has been trained on a public dataset [57] that accounts for different illumination conditions. Therefore, the failure modes identified can be mitigated with the help of the above-mentioned strategies.

The methods developed in this research account for some errors due to human demonstrations while providing guidelines to avoid errors that the method is not designed to handle, such as occlusion and suboptimal demonstrations. Each stage of this research, such as hand detection, hand tracking, robot hand–eye calibration, seam path generation, and robot welding path implementation, is independently developed, thereby ensuring a modular structure. Since HRI is an ongoing field of research, each modular stage can be updated with novel findings in the literature as future scope.

## 5. Conclusions and Future Work

In this research, we developed a novel end-to-end weld-seam detection system based on a human-hand tracking demonstration for robot welding operations. We developed methods to track the human hand using a tracking-by-detection approach with a trained hand-pose detection deep network. These tracking points are used to localise the weld seam, and subsequent image processing techniques were developed to isolate the weld seam edge. The weld seam edge is used to detect a continuous weld seam path using a search algorithm developed in this research. A thorough qualitative evaluation was conducted in which the rendered seam images were visually assessed. The accuracy of the weld seam in the image plane is ±1 pixel, and it is equivalent to ±1 mm in the physical space of our setup in more than 95% of the checkpoints. Line, curve, and saw-tooth weld seams were evaluated under varying environmental conditions, and the seam rendering on the image was observed within the bounds of the seam edge. Composite welding paths are also detected and evaluated. The weld path was projected to the robot coordinate space, and the implementation was evaluated on the UR10e robot.

The development of a human–robot interaction (HRI)-based method for robot welding has a potential impact on developing robots and interfaces that use human gestures as instructions. The robot implementation process in this research ensures path validation. However, there are different safety parameters to consider for real welding. Thus, future work can focus on developing safety measures after weld seam detection and during robot path planning. Additional safety procedures can also be developed for the methods presented in this paper to transform a semi-autonomous system into a fully automated system. Further investigation into mobile robot arms with hand-in-eye setups, along with different types of vision sensors such as active stereo, can make the robots more adaptive to different environmental conditions.

## Figures and Tables

**Table 1:** Evaluation of passive vision literature.

Papers	Category	Limitations
[11,12,13,14]	Active Vision	Moving laser parts. Requires laser scanning of the weld piece before seam detection. The moving part may impact the robot hand–eye calibration.
[15,16,18,30]	Passive Vision	Weldment must be placed in a limited space. Requires prior knowledge about the weldment type. Seam search algorithm requires the knowledge of the seam type.
[20,29,37,39]	Deep Learning	Requires large annotated data. Detection is related to the weld seam type in the dataset. Requires additional processing to determine the seam path.

**Table 2:** Results for seam path evaluation using user-defined seam path checkpoints.

Seam Type	|Γ|	$|\gamma_{0}|$	$r_{0}$	$\epsilon_{0}$	$|\gamma_{1}|$	$r_{1}$	$\epsilon_{1}$	$|\gamma_{2}|$	$r_{2}$	$\epsilon_{2}$
line	21	18	0.857	0.143	19	0.905	0.095	21	1.000	0.000
line	39	35	0.897	0.103	37	0.949	0.051	38	0.974	0.026
line	42	31	0.738	0.262	39	0.929	0.071	42	1.000	0.000
line	42	35	0.833	0.167	42	1.000	0.000	42	1.000	0.000
line	37	31	0.838	0.162	36	0.973	0.027	37	1.000	0.000
curve	34	25	0.735	0.265	33	0.971	0.029	34	1.000	0.000
curve	36	30	0.833	0.167	31	0.861	0.139	31	0.861	0.139
curve	44	42	0.955	0.045	44	1.000	0.000	44	1.000	0.000
curve	44	38	0.864	0.136	43	0.977	0.023	44	1.000	0.000
curve	35	31	0.886	0.114	34	0.971	0.029	34	0.971	0.029
saw	68	60	0.882	0.118	67	0.985	0.015	68	1.000	0.000
saw	38	33	0.868	0.132	35	0.921	0.079	37	0.974	0.026
saw	54	51	0.944	0.056	53	0.981	0.019	54	1.000	0.000
saw	59	55	0.932	0.068	57	0.966	0.034	57	0.966	0.034
saw	62	41	0.661	0.339	57	0.919	0.081	60	0.968	0.032

**Table 3:** A comparison of implementing A* search algorithm and Algorithm 2 on the weld seam.

	A* Search Algorithm					Algorithm 2				
Seam Type	Visited Nodes	Seam Nodes	Δ Nodes	Time (s)	Memory (MB)	Visited Nodes	Seam Nodes	Δ Nodes	Time (s)	Memory (MB)
line	226	158	68	0.011	6.825	223	158	65	0.016	4.793
line	197	163	34	0.009	6.684	198	163	35	0.016	4.68
line	257	194	63	0.011	6.684	234	194	40	0.019	4.671
line	221	181	40	0.01	6.702	222	181	41	0.018	4.69
line	257	203	54	0.011	6.693	238	203	35	0.023	4.683
curve	281	237	44	0.011	6.714	275	237	38	0.025	4.69
curve	248	185	63	0.011	6.722	242	185	57	0.022	4.708
curve	304	225	79	0.012	6.713	275	225	50	0.028	4.691
curve	264	217	47	0.011	6.733	265	217	48	0.026	4.712
curve	275	226	49	0.011	6.717	280	226	54	0.027	4.701
saw	383	348	35	0.016	6.739	365	348	17	0.043	4.709
saw	378	312	66	0.017	6.81	357	312	45	0.035	4.751
saw	429	346	83	0.02	6.812	397	346	51	0.053	4.748
saw	376	318	58	0.016	6.828	362	318	44	0.045	4.763
saw	323	319	4	0.014	6.806	323	319	4	0.035	4.749

**Table 4:** Results for mapping pixel coordinates to the robot coordinates. Robot coordinates are given in metres.

Group	*u*	*v*	*d*	$x_{r}$	$y_{r}$	$z_{r}$	Δx	Δy	Euclidean Distance
1	10	200	112	0.6706	−0.7244	0.0061	0.0009	0.0006	0.00107
	11	200	112	0.6715	−0.7238	0.0060			
2	300	250	113	0.9593	−0.5963	0.0014	0.0009	0.0006	0.00106
	301	250	113	0.9601	−0.5957	0.0014			
3	101	500	118	0.9294	−0.9185	0.0316	0.0008	0.0006	0.00102
	100	500	118	0.9286	−0.9190	0.0316			
4	351	600	118	1.1975	−0.8627	0.0247	0.0006	0.0009	0.00102
	351	599	118	1.1970	−0.8619	0.0247			
5	200	400	116	0.9578	−0.7818	0.0192	0.0006	0.0009	0.00207
	200	402	116	0.9589	−0.7835	0.0192			

## Data Availability

The original contributions presented in this study are included in the article. Further inquiries can be directed to the corresponding authors.

## Abbreviations

The following abbreviations are used in this manuscript:

BRIEFs	Binary Robust Independent Elementary Features
Cobot	Collaborative Robot
CAD	Computer-Aided Design
HRI	Human–Robot Interaction
LIDAR	Light Detection and Ranging
PHT	Probabilistic Hough Transform
PLA	Polylactic Acid
PNN	Probabilistic Neural Network
RGB	(Red Green Blue)
RGB-D	Red, Green, Blue, Depth
ROI	Region of Interest
ROS	Robot Operating System
SGBM	Semi-Global Block Matching
YOLO	You Only Look Once
